# Daily versus every other day oral iron supplementation in patients with iron deficiency anemia (DEODO): study protocol for a phase 3 multicentered, pragmatic, open-label, pilot randomized controlled trial

**DOI:** 10.1186/s40814-022-01042-y

**Published:** 2022-05-04

**Authors:** Amie Kron, M. Elisabeth Del Giudice, Michelle Sholzberg, Jeannie Callum, Christine Cserti-Gazdewich, Vidushi Swarup, Mary Huang, Lanis Distefano, Waseem Anani, Robert Skeate, Chantal Armali, Yulia Lin

**Affiliations:** 1 Precision Diagnostics and Therapeutics Program, Sunnybrook Health Sciences Centre, Toronto, M4N 3M5 Canada; 2 University of Toronto Quality in Utilization, Education and Safety in Transfusion (QUEST) Research Program, Toronto, Canada; 3 Department of Family and Community Medicine, Sunnybrook Health Sciences Centre, University of Toronto, Toronto, Canada; 4 Hematology Oncology Clinical Research Group, St. Michael’s Hospital, Toronto, Canada; 5 Li Ka Shing Knowledge Institute, St. Michael’s Hospital, Toronto, Canada; 6 Division of Hematology, Department of Medicine, St. Michael’s Hospital and University of Toronto, Toronto, Canada; 7 Department of Laboratory Medicine and Pathobiology, University of Toronto, Toronto, Canada; 8 Laboratory Medicine Program, University Health Network, Toronto, Canada; 9 Medical Services and Hospital Relations, Canadian Blood Services, Ottawa, Canada

**Keywords:** Iron deficiency, Iron deficiency anemia, Oral iron, Hemoglobin

## Abstract

**Background:**

Iron deficiency anemia (IDA) accounts for the majority of anemia cases across the globe and can lead to impairments in both physical and cognitive functioning. Oral iron supplementation is the first line of treatment to improve the hemoglobin level for IDA patients. However, gaps still exist in understanding the appropriate dosing regimen of oral iron. The current trial proposes to evaluate the feasibility of performing this study to examine the effectiveness and side-effect profile of oral iron once daily versus every other day.

**Methods:**

In this open-label, pilot, feasibility, randomized controlled trial, 52 outpatients over 16 years of age with IDA (defined as hemoglobin < 12.0 g/dL in females and < 13.0 g/dL in males and ferritin < 30 mcg/L) will be enrolled across two large academic hospitals. Participants are randomized in a 1:1 ratio to receive 300 mg oral ferrous sulfate (60 mg of elemental iron) either every day or every other day for 12 weeks. Participants are excluded if they are as follows: (1) pregnant and/or currently breastfeeding, (2) have a disease history that would impair response to oral iron (e.g., thalassemia, celiac disease), (3) intolerant and/or have an allergy to oral iron or vitamin C, (4) on new anticoagulants in the past 6 months, (5) received IV iron therapy in the past 12 weeks, (6) have surgery, chemotherapy, or blood donation planned in upcoming 12 weeks, (7) a creatinine clearance < 30 mL/min, or (8) hemoglobin less than 8.0 g/dL with active bleeding. The primary outcome is feasibility to enroll 52 participants in this trial over a 2-year period to determine the effectiveness of daily versus every other day oral iron supplementation on hemoglobin at 12 weeks post-initiation and side-effect profile.

**Discussion:**

The results of this trial will provide additional evidence for an appropriate dosing schedule for treating patients with IDA with oral iron supplementation. Additional knowledge will be gained on how the dosing regimen of oral iron impacts quality of life and hemoglobin repletion in IDA patients. If this trial is deemed feasible, it will inform the development and implementation of a larger multicenter definitive trial.

**Trial registration:**

ClinicalTrials.gov: NCT03725384. Registered 31 October 2018.

**Supplementary Information:**

The online version contains supplementary material available at 10.1186/s40814-022-01042-y.

WHO Trial Registration Dataset

**Table:** 

Data category	Information
Primary registry and trial identifying number	ClinicalTrials.gov: NCT03725384
Date of registration in primary registry	31 October 2018
Secondary identifying numbers	CTO 1534 version 5 09 July 2020
Source(s) of monetary or material support	University of Toronto, Alexandra Yeo Chair Grant in Benign Hematology Canadian Blood Services Transfusion Medicine Research Program Support Award
Primary sponsor	Sunnybrook Research Institute, 2075 Bayview Avenue, Toronto, Ontario, M4N 3M5
Contact for public queries	Yulia Lin, MD, FRCPC, CTBS 2075 Bayview Avenue, Room B204, Toronto, Ontario M4N 3M5 416-480-6100 ext. 2781 yulia.lin@sunnybrook.ca
Contact for scientific queries	Principal Investigator Yulia Lin, MD, FRCPC, CTBS Division Head, Transfusion Medicine and Tissue Bank, Precision Diagnostics and Therapeutics Program, Sunnybrook Health Sciences Centre Associate Professor, Department of Laboratory Medicine and Pathology, University of Toronto, 2075 Bayview Avenue, Room B204, Toronto, Ontario M4N 3M5 416-480-6100 ext. 2781 yulia.lin@sunnybrook.ca
Public title	Daily vs. every other day oral iron supplementation in patients with iron deficiency anemia (DEODO)
Scientific title	The frequency of oral iron supplementation in patients with absolute iron deficiency anemia: a pilot randomized controlled trial
Countries of recruitment	Canada
Health condition(s) or problem(s) studied	Iron deficiency anemia defined as hemoglobin less than 12.0 g/dL in females or less than 13.0 g/dL in males AND ferritin less than 30 mcg/L
Intervention(s)	Active comparator: ferrous sulfate 300 mg oral (60 mg of elemental iron) and vitamin C 500 mg every day × 12 weeks Control comparator: ferrous sulfate 300 mg oral (60 mg of elemental iron) and vitamin C 500 mg every other day × 12 weeks
Key inclusion and exclusion criteria	Ages eligible for study: ≥ 16 years Sexes eligible for study: both Accepts healthy volunteers: no Inclusion criteria: age ≥ 16 years, outpatients with iron deficiency anemia defined as hemoglobin less than 12.0 g/dL in females or 13.0 g/dL in males, AND ferritin less than 30 mcg/L Exclusion criteria: pregnancy; currently breastfeeding; known history of inflammatory disease, thalassemia or thalassemia trait, and inherited bleeding disorder; intolerance or lack of response to oral iron; 35 mg or more of elemental iron per day in 2 weeks prior to randomization; allergy to oral iron or vitamin C; intravenous iron therapy in the past 12 weeks; on new anticoagulant therapy initiated in the past 6 months (e.g., warfarin, apixaban, dabigatran, edoxaban, rivaroxaban); surgery, chemotherapy, or blood donation planned in upcoming 12 weeks; previously enrolled in the study; creatinine clearance less than 30 mL/min; hemoglobin less than 8.0 g/dL with active bleeding (defined as WHO grade-2 bleeding or higher in the past week)
Study type	Interventional Method of allocation: randomized, 1:1 ratio, computer-generated Masking: open label Framework: feasibility Primary purpose: treatment Phase III
Date of first enrollment	09 Jan 2019
Target sample size	Plan to enroll: 52 Participants enrolled: 52
Recruitment status	Enrollment complete
Primary outcome(s)	Outcome name: feasibility Metric: time to enroll 52 participants Time point(s) of primary interest: 2 years
Key secondary outcomes	Feasibility: proportion consenting to participate; proportion receiving allocated treatment; proportion completing laboratory tests, FACIT-fatigue scale, side effects questionnaire; adherence (time frame: 2 years; not designated as safety issue) Secondary Clinical Outcomes: mean hemoglobin increment at 4 and 12 weeks, proportion with complete hemoglobin response at 4 and 12 weeks, change in ferritin, serum iron and TSAT at 12 weeks, quality of life (FACIT-fatigue scale) at 4, 8, and 12 weeks, gastrointestinal adverse effects at 4, 8, and 12 weeks, need for escalation in therapy, and proportion with a drop in hemoglobin of 1.0 g/dL or more at 4 and 12 weeks (time frame: 2 years; not designated as safety issue)
Ethics review	Status: approved Date of approval: 25 Oct 2018 — Sunnybrook Health Sciences Centre; 05 Apr 2019 — St. Michael’s Hospital Name and contact details of ethics committees(s): ○ Research Ethics, Human Protections Program, Sunnybrook Health Sciences Centre, 2075 Bayview Avenue, Room C823 or C827, Toronto, Ontario, M4N 3M5, 416-480-6100 ext. 88144 • Unity Health Toronto Research Ethics Board, St. Michael’s Hospital, 30 Bond St, Toronto, Ontario,M5B 1W8, 416-864-6060 ext. 42557

## Introduction

### Background and rationale

Iron deficiency anemia (IDA) is a global health problem and the most common cause of anemia worldwide [1]. There is a significant variation in IDA based on geographical location and population studies. In North America and Europe, its prevalence ranges from 1 to 2% in men [2], 2–5% in females [2–5], 9–11% in adolescent females [6], and 17–31% [3, 7] in pregnancy. Iron is an important nutrient for the production of red blood cells but also has many biological functions including energy production, DNA synthesis, and cell proliferation [8]. Patients with iron deficiency (ID) and IDA can present with a multitude of symptoms including fatigue, restless leg syndrome [9], and pica [10]. In addition, IDA can lead to impacts on physical function such as diminished aerobic capacity (reduce VO_2_ max), work intolerance, and fatigue [11] and cognitive impairments in attention, memory, speed, and executive planning function [12–14].

The World Health Organization (WHO) defines anemia as a hemoglobin less than 12.0 g/dL for females and less than 13.0 g/dL for males [15]. Although the definition of anemia is well accepted, the definition of IDA is less standardized. Based on a systematic review conducted by Guyatt et al. to determine the diagnostic value of laboratory tests in the diagnosis of ID, serum ferritin was found to be the most predictive and a better diagnostic test than mean cell volume (MCV) and transferrin saturation (TSAT) [16]. Serum ferritin values between 15 and 25 mcg/L were associated with a likelihood ratio of 8.83 for ID. Goodnough et al. found that a serum ferritin of less than 30 mcg/L was associated with a sensitivity of 92% for ID and a positive predictive value of 83% [17]. To ensure a population with a high likelihood of IDA for this study, ID has been frequently defined as a serum ferritin of less than 30 mcg/L [8, 18, 19].

Oral iron supplementation is associated with increasing hemoglobin in multiple studies in females, pregnancy, and elderly patients [20–22]. The most common oral iron supplements currently used are ferrous gluconate, sulfate, and fumarate [8]. Ferrous formulations have greater bioavailability than ferric preparations since the ferrous form is more readily absorbed by enterocytes. These supplements are inexpensive and often covered by health insurance plans. Side effects of oral iron supplementation include constipation, nausea, diarrhea, abdominal pain, vomiting, heartburn, flatulence, and dark stools [8, 23]. When compared to placebo in randomized controlled trials, ferrous sulfate had an increased incidence of gastrointestinal (GI) side effects, although there did not appear to be a significant association between GI side effects and dose [23]. Although intravenous iron is also available, it has been associated with rare serious infusion side effects including anaphylaxis, limited resources for infusion facilities, and nursing. Thus, oral iron supplementation should be the first line of therapy when time permits [18, 24, 25].

The optimal dose and frequency of oral iron supplementation still remains unclear. A recent systematic review of iron supplementation in females between menarche and menopause showed that intermittent dosing reduced the risk of anemia (RR 0.65, 95% *CI* 0.49 to 0.87) and improved hemoglobin compared with placebo [26]. Intermittent dosing was similar to daily dosing in effect on anemia and hemoglobin but had lower ferritin improvement as well as having fewer side effects (RR 0.41, 95% *CI* 0.21 to 0.82) compared with daily dosing [26]. Two different dosing regimens were investigated by Stoffel et al. in females aged 18 to 45 with a serum ferritin less than 25 mcg/L who did not have anemia [27]. It was concluded that single-dose oral iron supplements on alternate days optimize iron absorption and may therefore be the preferred dosing regimen. The proposed study attempts to determine the feasibility of enrolling 52 participants in a pilot, randomized controlled trial (RCT) over a 2-year period.

### Objectives

The primary objective of this study is to determine the feasibility of performing a pragmatic, open-label, RCT to evaluate effectiveness of oral ferrous sulfate 300 mg (60 mg elemental iron) once daily versus every other day to improve hemoglobin at 12 weeks post-initiation. The secondary purpose of this trial is to determine the effectiveness and side-effect profile of alternative oral iron supplementation dosing regimens on IDA, including hemoglobin.

### Trial design

**Fig. 1:**
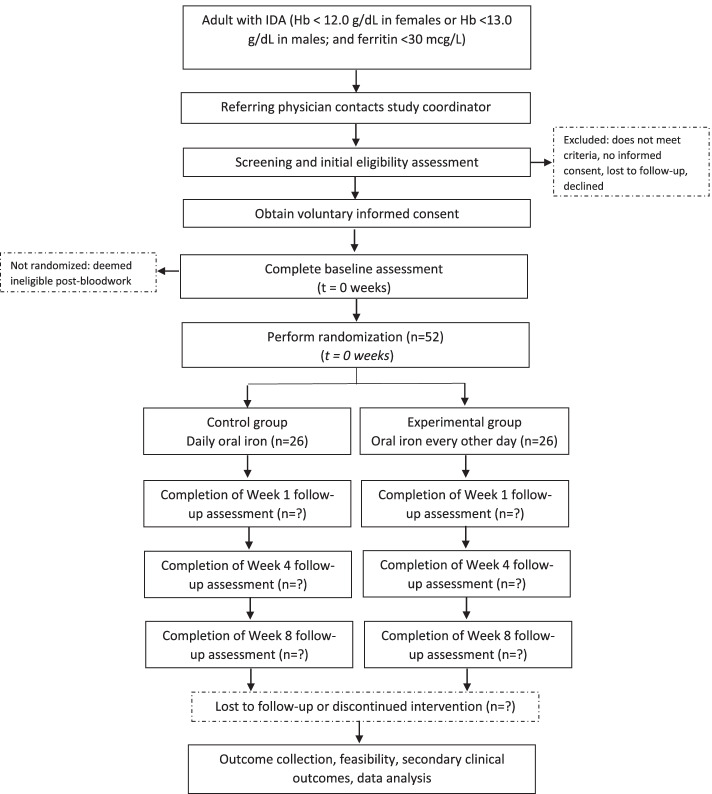
Pilot trial schematic

This is a pilot, pragmatic, open-label, RCT in outpatients with IDA. A total of 52 participants will be randomized in a 1:1 ratio to receive oral iron either every day or every other day. Figure 1 shows the schematic of this pilot trial design. The Standard Protocol Items: Recommendations for Interventional Trials (SPIRIT) checklist is provided in Additional file 1.

## Methods

### Participants, interventions, and outcomes

#### Study setting

This study is taking place at two large academic hospitals in Toronto, Canada, affiliated with the University of Toronto: Sunnybrook Health Sciences Centre and St. Michael’s Hospital, Unity Health Toronto.

#### Eligibility criteria

The eligibility criteria of this study are broad to increase the generalizability and feasibility of the proposed trial. The exclusion criteria are predominantly conditions where oral iron has already been shown to be ineffective. There are no exclusions based on sex, race, or ethnicity in this trial.

### Inclusion criteria

1. Age ≥ 16 years
2. Outpatients with iron deficiency anemia defined as hemoglobin less than 12.0 g/dL in females or less than 13.0 g/dL in males AND ferritin less than 30 mcg/L.

### Exclusion criteria

1. Pregnancy and/or currently breastfeeding
2. Known history of inflammatory bowel disease, celiac disease, thalassemia or thalassemia trait, and/or inherited bleeding disorder
3. Known intolerance or lack of response to oral ferrous gluconate, sulfate, or fumarate in the last 12 weeks
4. Multivitamin and mineral supplements (35 mg or more of elemental iron per day) in 2 weeks prior to randomization
5. Allergy to oral iron
6. Allergy to any of the following medicinal and non-medicinal ingredients in ferrous sulfate: ferrous sulfate, calcium citrate, crospovidone, FD&C Red #40-Aluminum Lake, FD&C Yellow #6-Aluminum Lake, magnesium stearate, microcrystalline cellulose, polyethylene glycol, polyvinyl alcohol, purified water, and talc titanium dioxide
7. Allergy to any of the following medicinal and non-medicinal ingredients in vitamin C: ascorbic acid, colloidal silicon dioxide, croscarmellose sodium, magnesium stearate, microcrystalline cellulose, and stearic acid
8. Intravenous iron therapy in the past 12 weeks
9. On new anticoagulant therapy initiated in the past 6 months (e.g., warfarin, apixaban, dabigatran, edoxaban, rivaroxaban)
10. Surgery, chemotherapy, and/or blood donation planned in upcoming 12 weeks
11. Previously enrolled in the study
12. Creatinine clearance less than 30 mL/min
13. Hemoglobin less than 8.0 g/dL with active bleeding (defined as WHO grade-2 bleeding or higher in the past week)

### Consent

Patients interested in participating and meeting inclusion and exclusion criteria are given an informed consent form (Additional file 2). The study coordinator conducts an informed consent discussion which includes the rationale for the study, the anticipated risks and benefits of participation, and their rights as a study participant (including withdrawal at any time). Capable participants are offered an opportunity to ask questions and consult with their family and/or their primary care physician before enrollment. If the patient is deemed incapable of providing informed consent for the study, study personnel approach the patient’s physician to determine if it is safe to delay treatment while a substitute decision-maker is identified and approached. As the initiation of oral iron is not urgent, there is enough time to assess the patient for inclusion in the study before initiating treatment. The subject is invited to participate and must provide written informed consent prior to any study-related procedures. Remote consent using a certified signature software (e.g., Adobe Sign) is permitted; this change was made as a result of the COVID-19 pandemic. Once the subject is enrolled, his/her family physician is informed about his/her participation in the study. If the participant does not wish to participate, the reason for declining is documented. All sites obtained Research Ethics Board (REB) approval of the protocol and informed consent form before commencing any study activities.

### Interventions

Open-label oral ferrous sulfate-300 mg (60 mg of elemental iron; JAMP, Boucherville, Québec, Canada) and vitamin C 500-mg tablets (WAMPOLE, Boucherville, Québec, Canada) are the natural health products used throughout this study. Once enrolled, participants are randomized to either the daily or every other day treatment arm. Randomization occurs within 1 week of enrollment. Once randomization occurs, a prescription for the dosing regimen is given to the participant to bring to the pharmacy at the local study site. Participants opting for virtual clinical visits may receive their study drug by courier. As participants receive the total treatment for 12 weeks at once, they were notified by the research coordinator that it was their responsibility to not share the tablets with others. The consent form warned participants that the pills should be kept away from children as a child taking an excessive amount of tablets would need to seek prompt medical attention.

#### Daily dosing

Starting on day 1, participants take ferrous sulfate on an empty stomach with vitamin C daily for a period of 12 weeks. Participants are instructed to take oral iron at bedtime but can take it at another time during the day if more convenient. If participants cannot tolerate the oral iron on an empty stomach, they are instructed to take it with a small amount of food and avoid eating dairy, antacids, calcium, and tannins (e.g., coffee/tea) at the same time.

#### Every other day dosing

Starting on day 1, participants take ferrous sulfate on an empty stomach with vitamin C every other day for a period of 12 weeks. Participants following this dosing schedule are instructed to take the tablets in the same manner as those on the daily dosing schedule.

#### Packaging

The oral ferrous sulfate tablets are packaged in bottles containing the corresponding amount of pills depending on which treatment arm the patient has been assigned to. One patient kit contains a bottle of oral ferrous sulfate and another bottle of vitamin C to be taken together.

#### Modifications

Participants are followed until 12 weeks or until there is an escalation in therapy as defined by the need for the following: alternative oral iron therapy (including if a participant in the every other day group escalates to daily iron), need for intravenous iron, need for transfusion, or a visit to the emergency department related to anemia. At the week 4 assessment, participants with a drop in hemoglobin of more than 1.0 g/dL from baseline are referred back to the most responsible physician (MRP) for management and therapy escalation. It is anticipated that approximately 30% of participants may not tolerate oral iron. Side effects may improve over the initial 1–2 weeks of intervention. In some cases, participants may not be able to tolerate the intervention and, in such cases, are instructed to follow their planned schedule as prescribed. If they continue to have symptoms, participants are instructed to step down in frequency (e.g., from daily to every other day, from every other day to two times per week, from two times per week to once per week) and to record how frequently they are able to take oral iron. If participants are unable to take the oral iron at all, they continue to be followed as long as they do not meet any of the following criteria for withdrawal: become pregnant during the course of the study, any deviation from inclusion/exclusion criteria, and serious adverse events.

#### Adherence

Throughout the study duration, participant compliance with ingesting oral iron is monitored through an adherence diary, direct contact between the patient and study coordinator at designated assessment times (i.e., 1, 4, 8, and 12 weeks post-initiation), and a pill count following the 12-week supplementation period. Participants check off when they have taken their tablet on the diary calendar (provided in paper format), which indicates which days they should be ingesting oral ferrous sulfate. At the 1st, 4th, and 8th weeks after beginning the study treatment, participants are contacted (telephone or email) to assess adherence. If a participant misses a pill, they are advised to continue with the next dose as directed on the calendar and not to double up on dosing. At the end of the 12-week supplementation period, the participant returns the adherence diary, the pill bottle provided to them, and any unused tablets for a final pill count; this number will be compared to the number of tablets the participant should have ingested during the 12 weeks. Participants opting for remote assessments are requested to courier their pill bottles and adherence diary back to the local site, in which case a prepaid return envelope is provided to participants by the study site.

#### Prior and concomitant medications

Throughout the study duration, and 2 weeks leading up to randomization, participants are not permitted to receive other sources of iron supplementation, including, but not limited to, other oral ferrous tablets and intravenous iron therapy. Participants are advised not to take oral iron within 2 h of antibiotics, bisphosphonates [28], levodopa, methyldopa [29], mycophenolate mofetil [30], or thyroid medication [31]. To allow tracking of confounders, participants are asked to notify the study coordinator if they choose to take part in another intervention during the study.

### Outcome measures

#### Feasibility outcome measures

The primary feasibility outcome of the trial is enrollment defined as documentation of informed consent and confirmation of eligibility. If the study is unable to enroll 52 participants in a 2-year period, the study as it is currently designed will not be deemed feasible. To determine feasibility of this study, the following proportions will be evaluated at the end of the trial: (1) eligible participants consenting to participate and receiving the allocated treatment; (2) treated participants completing laboratory tests, side-effect questionnaire, and the Functional Assessment of Chronic Illness Therapy (FACIT)-fatigue scale; (3) treatment doses taken as per protocol based on pill count; (4) participants taking at least 90% of their prescribed doses; and (5) participants requiring a step down in therapy. Methods of aggregation and specific measurement time points of interest for analysis are outlined in Table 1.

**Table 1:** Methods of aggregation for feasibility outcome measures

Proportion	Method
Eligible participants consenting to participate	$\frac{\#\;\text{meeting}\;\text{eligibility}\;\text{criteria}\;-\;\#\;\text{in}\;\text{whom}\;\text{consent}\;\text{not}\;\text{obtained}}{\#\;\text{meeting}\;\text{eligibility}\;\text{criteria}}$
Consenting participants receiving the allocated treatment	$\frac{\#\;\text{consenting\;to\;treatment}\;-\;\#\;\text{not\;administered\;allocated\;treatment}}{\#\;\text{consenting\;to\;treatment}}$
Treated participants completing 4- and 12-week laboratory tests	$\frac{\#\;\text{completing}\;100\%\;\text{of\;weeks}\;4\;\text{and}\;12\;\text{lab\;tests}}{\#\;\text{consenting\;to\;treatment}}$
Treated participants completing 4-, 8-, and 12-week side effect questionnaire	$\frac{\#\;\text{completing}\;100\%\;\text{of\;weeks}\;4,\;8,\;\text{and}\;12\;\text{side-effect\;questionnaire}}{\#\;\text{consenting\;to\;treatment}}$
Treated participants completing 4-, 8-, and 12-week FACIT-fatigue scale	$\frac{\#\;\text{completing}\;100\%\;\text{of\;weeks}\;4,\;8,\;\text{and}\;12\;\text{FACIT-fatigue\;scale}}{\#\;\text{consenting\;to\;treatment}}$
Treatment doses taken as per protocol based on pill count	$\frac{\text{Total}\;\#\;\text{of\;doses}\;-\;\#\;\text{of\;missed\;doses}}{\text{Total}\;\#\;\text{of\;doses}}$
Treated participants taking at least 90% of their prescribed doses	$\frac{\#\;\text{of\;participants\;taking\;at\;least}\;90\%\;\text{of\;prescribed\;doses}}{\text{Total}\;\#\;\text{of\;treated\;participants}}$
Treated participants requiring a step down in therapy	$\frac{\#\;\text{of\;participants\;requiring\;a\;step\;down\;in\;therapy}}{\text{Total}\;\#\;\text{of\;treated\;participants}}$

### Secondary clinical and safety outcome measures

#### Laboratory assessments

Hemoglobin increments will be calculated from the hemoglobin levels at 4 and 12 weeks minus the baseline hemoglobin value. A complete hemoglobin response will be defined as the proportion of participants with a hemoglobin greater than or equal to 12.0 g/dL in females and 13.0 g/dL in males at 4 and 12 weeks. Change in reticulocyte count at 4 and 12 weeks will be defined as the 4- or 12-week reticulocyte count minus the baseline count. Change in ferritin, serum iron, and TSAT at 12 weeks will be defined as the value at 12 weeks minus the baseline value.

#### Quality-of-Life (FACIT-fatigue scale)

Given the impacts of IDA extend beyond anemia and can affect an individual’s physical, emotional, and social well-being, quality-of-life measures are important to incorporate in the current study. The FACIT-fatigue scale is administered to participants at the 4-, 8-, and 12-week assessment to evaluate anemia-related fatigue [32]. The scale is administered by the study coordinator and consists of 13 patient-reported items with a 7-day recall period. Items are scored from 0 (not at all) to 4 (very much so).

#### Side-effect questionnaire

The side effects of oral iron can have a significant impact on adherence with studies reporting up to 40% nonadherence [33]. A recent study specifically developed a one-page side-effect questionnaire in patients on oral iron supplementation [34]. A modified version of this questionnaire is used in the current study to assess the proportion of participants with side effects at 4, 8, and 12 weeks. The proportion of participants who stop taking oral iron due to side effects at 4 and 12 weeks is also documented.

#### Need for escalation of therapy

At the 12-week assessment, participants are assessed for the need for escalation in therapy defined as an increase in oral iron regimen from every other day to daily, need for intravenous iron, or a visit to an emergency department related to anemia. The proportion of participants with a drop in hemoglobin of 1.0 g/dL of more at weeks 4 and 12 from baseline is also recorded. Patients with unexplained iron deficiency or ongoing bleeding are referred by the study doctor for further assessment (e.g., gastroenterologist, gynecologist).

### Participant timeline

The participant timeline is described in Table 2.

**Table 2:**
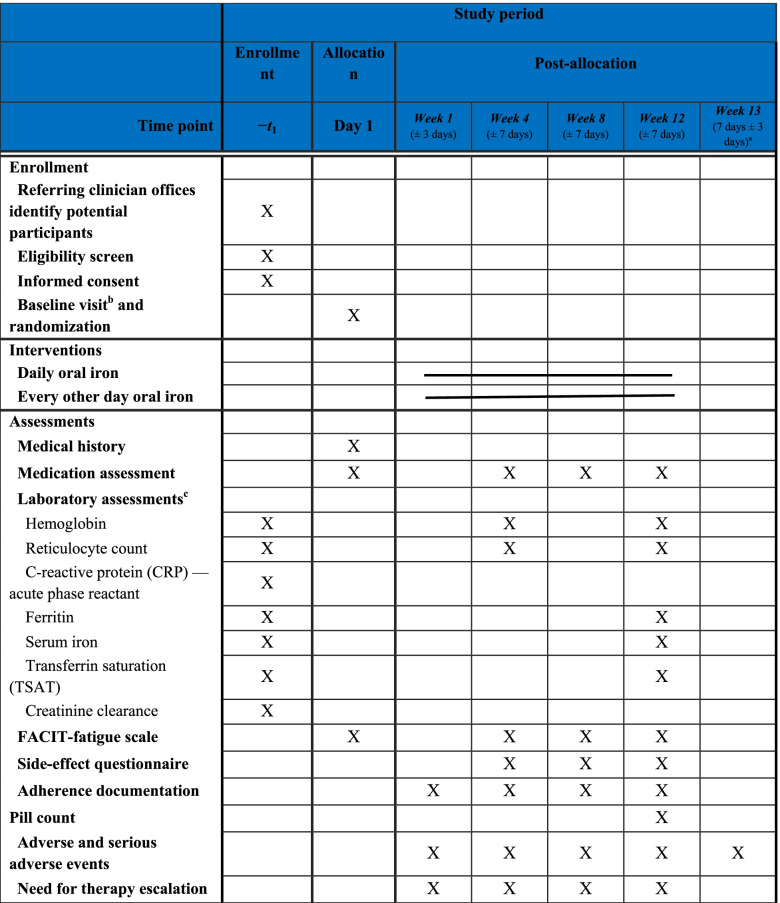
Schedule of enrollment, interventions, and assessments/procedures

^a^After the week 12 final study visit

^b^Baseline assessments may be performed in person or remotely (telephone/Zoom)

^c^Baseline labs must be completed within 2 weeks of randomization, with creatinine values within 3 months of randomization

### Sample size

The sample size of the study is based on an anticipated enrollment of 26 patients per year at the two sites combined. With a sample size of 52, we will be able to estimate a completion rate for laboratory tests and questionnaires and an adherence rate (participants taking at least 90% of their prescribed doses) of 90% to within a 95% confidence interval of +/−8%. For participants requiring a step down in therapy, we will be able to estimate a rate of 5% to within a 95% confidence interval of +/−6%.

### Recruitment

Recruitment will occur for the duration of 2 years. Participants meeting inclusion criteria are identified through referring clinician offices (hematology and primary care physicians). With permission from the MRP to enter the patient’s circle of care, the study coordinator approaches potentially eligible participants. The study coordinator introduces the trial, confirms eligibility, and conducts the informed consent discussion. Study personnel screen participants for exclusion criteria by asking about the patient’s medical history and reviewing their bloodwork that will be taken as a standard of care when visiting the MRP.

Another recruitment site is the Canadian Blood Services (CBS). Toronto area blood donors that have been deferred due to low hemoglobin levels are provided with a letter explaining that the donor was deferred and for the donor to visit their family physician for further investigation. In addition, a letter to the family physician explains key points about the study purpose, rationale, procedures, and detailed information about the trial. If the patient is interested in participating, their physician can refer the patient to one of the study sites. It is also acceptable for the patient to contact the study site directly in which case permission from the MRP is not required.

### Assignment of interventions

#### Allocation, concealment, and implementation

Participants are randomized in a 1:1 ratio according to a computer-generated allocation sequence to receive either once daily dosing or every other day dosing. The randomization code is generated in random blocks of 4 to 6, stratified by center and baseline hemoglobin (hemoglobin ≥ 10 g/dL *OR* < 10 g/dL). Randomization is provided by the Centre for Clinical Trial Services at Sunnybrook Health Sciences Centre to the sites in opaque numbered envelopes to ensure concealment and no members of the study team have access to the allocation scheme. The envelope is only opened by the study coordinator once baseline measurements have been completed and the participant has been enrolled in the trial. Following randomization, participants receive oral iron tablets for a period of 12 weeks, with clinical and subjective data collection occurring 1, 4, 8, and 12 weeks following commencement of oral iron (see Fig. 1). The study aims to recruit 52 participants in total, randomizing 26 participants to each study arm.

#### Blinding

This study is an open-label trial. The statistician will remain blinded when performing the final analysis.

### Data collection, management, and analysis

#### Data collection methods

Source documents have been developed for each assessment throughout the study and are used by study personnel to complete case report forms (CRFs). Demographic and medical history data are collected at the baseline visit using a baseline assessment questionnaire (Additional file 3) and participant medical records. Dietary assessments (e.g., vegetarian, vegan) were done at the baseline visit to allow for an estimate of dietary iron intake. For female participants where menorrhagia is the cause of iron deficiency anemia, a pictorial blood assessment chart [35, 36] is used to assess the level of menstrual bleeding. Laboratory results are reviewed by the principal investigator and assessed for clinical significance for results considered out of range. Participants may obtain bloodwork at the local study site or at a local blood lab; the same laboratory must be used for the duration of the study. Paper patient adherence diaries are used to assess adherence and photos of the calendar e-mailed at the follow-up visits to the study coordinator. Adverse events and quality of life are assessed using the side-effect questionnaire and FACIT-fatigue scale, respectively. A medication log is used throughout the study to confirm if a participant has started any new or discontinued medications. Participants will also be compensated for parking expenses incurred if they choose to have their follow-up assessments and/or bloodwork completed at the local study site. Intervention discontinuation, withdrawal of consent, and participants lost to follow-up are documented.

#### Data management

Site research coordinators are responsible for document management and database development and management. Study data from original study forms and patient surveys are entered and maintained on a secure password-protected database developed using REDCap [37, 38] located at Sunnybrook Health Sciences Centre, Toronto, Ontario, Canada. REDCap is accessible to study team members only for data entry purposes. Each center enters data for the study participants enrolled at their site, and quality control oversight is done by the research coordinator located at the coordinating center (Sunnybrook Health Sciences Centre). The study coordinator reviews the electronic data for all participants on a regular basis for completeness and consistency. Quality and completeness of data entry are reviewed as soon as possible after data entry. Data integrity is enhanced through controls that require entry of valid data types and, where applicable, values within expected reference ranges. Data queries generated by identification of incomplete or inconsistent data is raised and resolved by the study coordinator or principal investigator in an ongoing manner. Corrections or changes to the data set are tracked by the data management system. Data does not continue to be collected for participants discontinuing the intervention. For participants deviating from intervention protocols, data is not collected for those agreeable to proceed with study participation.

### Statistical methods

All analyses will be conducted by MacroStat Inc. using Statistical Analysis Software (v9.4 for Windows, Cary, NC) and R package (v3.6.1). For the final analysis plan, point estimates of feasibility events, including enrollment, adherence to protocol and accrual, will be presented as proportions with 95% confidence intervals. Continuous data will be presented as means and standard deviations, or medians and interquartile ranges, as appropriate. An intention-to-treat analysis will be performed on all participants randomized for the secondary efficacy outcome. A per-protocol analysis will be conducted for participants who have taken at least 90% of the prescribed doses. Subgroup analysis will be performed on the stratified groups with hemoglobin 10.0 g/dL or greater and hemoglobin less than 10.0 g/dL. Additional subgroup analysis will include the following: participants with ongoing bleeding (WHO grade 2 or higher) versus no bleeding and normal vs. increased C-reactive protein (based on the normal range). Given that the current study is a feasibility trial, sex and gender differences are not being accounted for.

### Monitoring

#### Data monitoring

A data monitoring committee is not deemed to be required for this study because of the following: (1) this is a pilot randomized trial to assess feasibility, (2) there are no concerns regarding unacceptable toxicity, (3) the experimental arm is the same drug but at a lower dose, (4) the primary outcome is not a “major endpoint” such as mortality, (5) there are no ethical concerns regarding possible extreme efficacy of one arm so as to consider stopping the trial early, and (6) the study investigators are able to perform a safety assessment. Safety and interim analyses will be performed by an independent statistician blinded to treatment allocation after enrolling 20 participants into the study. The principal investigator will stop the study prior to its completion if difficulty in study recruitment or retention significantly impacts the ability to evaluate study endpoints, or any new information becomes available during the trial that necessitates stopping the study.

#### Harms

All adverse events (AE) are documented and assessed for relatedness from 1 week from baseline up to 1 week after the final study visit. Investigations into potential AEs are done during each contact with the participant using the side-effect questionnaire. All AEs are recorded on the eCRF and coded as per the Common Terminology Criteria for Adverse Events (v4.0). Study investigators will report serious adverse events (SAEs) to the sponsor within the following timelines: all deaths and immediately life-threatening events, whether related or unrelated, within 24 h of site awareness, and SAEs other than death and immediately life-threatening events, regardless of relationship, within 72 h of site awareness.

#### Auditing

Independent monitoring for this study is the responsibility of the sponsor, Sunnybrook Research Institute, and occurs both in person and remotely. A monitoring plan has been developed to outline the frequency of monitoring, monitoring procedures, the level of site monitoring activities, and the distribution of monitoring report. The monitor performed a site initiation visit for all sites prior to study commencement to ensure sites were prepared to conduct study procedures to institutional, provincial, and Health Canada standards. Monitoring visits involve reviewing study procedures, participant data, and Health Canada regulation compliance. After each visit, reports are generated to address any changes that need to be made or considered for appropriate regulatory compliance and patient safety.

## Discussion

The results of this trial will inform the feasibility of performing a larger multicenter trial evaluating the optimal oral iron supplementation dosing schedule for adult patients with IDA. Since the start of the trial, results from two recent RCTs support an alternate-day dosing schedule to maximize iron absorption in anemic adults [39, 40]. In one study, the median hemoglobin rise in 62 anemic adults supplemented with 120 mg of elemental iron on alternate days did not significantly differ from patients receiving 60 mg of iron twice daily, although those in the twice-daily group reported more GI side effects (*p* = 0.03) [39]. In another study of 40 patients, mean increases in hemoglobin were significantly higher in IDA patients receiving a single dose of 60 mg ferrous sulfate on alternate days (1.59 ± 0.53 gm/dl) compared to the everyday group (0.41 ± 0.25 gm/dl, *p* < 0.005), and the supplementation was also better tolerated in the alternate-day group [40]. Neither study reported on quality-of-life outcomes. Although there is increasing support for an intermittent dosing schedule for oral iron in IDA patients [41], additional studies such as the current RCT are needed to understand how hemoglobin repletion, adverse effects, and quality of life vary in patients according to the dosing regimen.

Ferrous sulfate was chosen as the elemental iron product for this study as it has been shown to be superior to other oral iron supplements and well tolerated [22, 42]. When compared to iron polysaccharide complex drops, infants and children supplemented with ferrous sulfate daily had a higher resolution of IDA (29% vs. 6%; *p* = 0.04) and reported less GI side effects (*p* = 0.04) [42]. Females with IDA treated with oral ferrous sulfate tablets have also shown mean hemoglobin increases significantly greater than a group supplemented with ferric preparations (i.e., ferric protein succinylate tablets) [22]. Ferrous iron compounds also have high bioavailability compared to the ferric form [43] and are less costly than other forms of oral iron supplementation. This trial included vitamin C as an adjunct to increase iron absorption, although more recent data published after the initiation of the trial suggest that there may not be benefit [44]. In a definitive trial, vitamin C will be removed, and participants will only take oral iron supplements. To focus the study on adult patients, participants in the study are greater than 16 years of age. Pregnant females and/or those who are currently breastfeeding are excluded from this study as hepcidin concentrations, a regulator of the bioavailability of iron, are known to be altered during this period [45]. Participants with inflammatory bowel disease, celiac disease, bleeding disorders, and/or nephritis were excluded as these comorbidities can also increase hepcidin concentrations and subsequently impair iron absorption. In a definitive trial, it may be important to capture the timing of the oral iron intake as hepcidin has a diurnal variation which may impact absorption.

Given that the trial design requires frequent follow-up assessments and bloodwork, several strategies were employed to promote participant recruitment, safety, and adherence. This was especially important once the trial was restarted during the COVID-19 pandemic. The primary strategy was amending the protocol to allow for all aspects of the participants involvement in the trial to occur virtually. Specifically, baseline and week 12 follow-up consult appointments with the study investigator can occur virtually (telephone, Ontario Telehealth Network, Zoom) as a physical examination is not necessary, and all follow-up assessments are done with the study coordinator by telephone. Study tablets are filled at the study site’s local pharmacy and couriered to the participant with their adherence diary. To improve the convenience of returning study materials at the end of the study, pre-paid return envelopes for the pill bottles and original adherence calendar are also provided in the initial shipment. Documenting adherence and assessing participants for intolerable side effects warranting dose modification required the study design to be open label. The authors recognize this may introduce bias which will be mitigated by ensuring the statistician and investigators are blinded to treatment allocation during the analysis phase. Bloodwork completed at a local lab was also permitted to improve participant comfort by limiting entry into study hospitals during the COVID-19 pandemic.

Implementing this trial using an entirely virtual platform has expanded our recruitment pool to participants outside of the regional catchment area of the study hospitals. Additional recruitment strategies include engaging the local site’s family practice team and collaborating with Canadian Blood Services to identify eligible participants. Engaging family practice facilitates identification of patients with IDA who have not yet been prescribed a treatment regimen for anemia and may potentially be eligible for the trial. Prior to donation at Canadian Blood Services, donor hemoglobin is measured. The eligible hemoglobin for donation is 12.5 g/dL in females and 13.0 g/dL in males; donors not meeting these criteria are therefore easily identified for participation in the trial. The authors are cognizant that strict cutoffs for hemoglobin were used to identify eligible participants. Recently, researchers have argued that the use of sex-specific reference ranges lead to inequities in the diagnosis and treatment of anemia, leaving a higher proportion of women than men with their anemia inappropriately managed [46, 47]. The authors will define anemia using hemoglobin thresholds that consider physiological differences in sex in a future definitive RCT. In addition, other indices of iron deficiency such as the reticulocyte hemoglobin will be considered in the future [48].

Overall, IDA is a common and prevalent condition with potential adverse consequences if left untreated. This pilot, pragmatic, open-label RCT aims to optimize effectiveness of oral iron supplementation while minimizing side effects to improve treatment for patients. Because IDA is a global health problem, common in clinical practice and treatable, this study, although simple in its question and design, will have a significant practical impact on how clinicians treat outpatients with IDA and how patients tolerate therapy. If the pilot trial is deemed to be feasible, a definitive multicenter trial will be planned.

### Protocol amendments

All protocol amendments are submitted to Clinical Trials Ontario and/or Health Canada (where applicable) and only implemented as per guidelines of these regulatory bodies. Modifications to the study methods will be reported in the final study trial report. A summary of changes document is retained to track all protocol versions and amendments.

### Confidentiality

Information about study participants is kept confidential and managed according to the requirements of the Personal Health Information Act of 2004 (PHIPA) and the Research Ethics Boards. Each participating site maintains appropriate medical and research records for this study, in addition to regulatory and institutional requirements for the protection of confidentiality of participants. All source documents containing personal health information are de-identified using a unique study identifier and stored in a secured locked filing cabinet at the study site. Study documents with identifying information such as referral forms and informed consent forms are stored in separate study binders, and all electronic study files are stored on a password-protected network drive at the study site. Only investigators and research team members listed on the task delegation log have access to participant medical records and collect only the information needed for the study. Sponsor-delegated monitors, representative of institutional committees, and regulatory authority representative of the country in which the study is being conducted also have access to examine records for the purposes of quality assurance reviews, audits, and evaluation of study safety and progress. The REB, investigator, and regulatory sponsor will retain study essential documents as per local regulatory requirements and GCP guidelines. Essential documents will be maintained in a secure and confidential manner for participating sites for a period of 25 years and then destroyed according to local and national policy and requirements. Only de-identified data will be transmitted to the statistician for data analysis purposes.

### Ancillary and post-trial care

Participants are not compensated for participating in the trial.

### Dissemination policy

Results of the study will be made available online through ClinicalTrials.gov, published in peer-reviewed journals, and shared with clinicians and stakeholders through presentations at local, national, and international meetings and conferences. All presentations and manuscript drafts will be reviewed by the authors of the current protocol prior to submission. Criteria for authorship was based on recommendations by the International Committee of Medical Journal Editors.

## Supplementary Information

**Additional file 1.**

## Data Availability

The datasets used and/or analyzed for this study will be available from the corresponding author upon reasonable request.

## Abbreviations

AE: Adverse event
CRF: Case report form
FACIT: Functional Assessment of Chronic Illness Therapy
GI: Gastrointestinal
ID: Iron deficiency
IDA: Iron deficiency anemia
MCV: Mean cell volume
MRP: Most responsible physician
RCT: Randomized controlled trial
REB: Research ethics board
SAE: Serious adverse events
TSAT: Transferrin saturation
WHO: World Health Organization
